# Prisoners in Their Habitat? Generalist Dispersal by Habitat Specialists: A Case Study in Southern Water Vole (*Arvicola sapidus*)

**DOI:** 10.1371/journal.pone.0024613

**Published:** 2011-09-09

**Authors:** Alejandro Centeno-Cuadros, Jacinto Román, Miguel Delibes, José Antonio Godoy

**Affiliations:** 1 Department of Conservation Biology, Estación Biológica de Doñana, Consejo Superior de Investigaciones Científicas, Sevilla, Spain; 2 Department of Integrative Ecology, Estación Biológica de Doñana, Consejo Superior de Investigaciones Científicas, Sevilla, Spain; Monash University, Australia

## Abstract

Habitat specialists inhabiting scarce and scattered habitat patches pose interesting questions related to dispersal such as how specialized terrestrial mammals do to colonize distant patches crossing hostile matrices. We assess dispersal patterns of the southern water vole (*Arvicola sapidus*), a habitat specialist whose habitat patches are distributed through less than 2% of the study area (overall 600 km^2^) and whose populations form a dynamic metapopulational network. We predict that individuals will require a high ability to move through the inhospitable matrix in order to avoid genetic and demographic isolations. Genotypes (N = 142) for 10 microsatellites and sequences of the whole mitochondrial Control Region (N = 47) from seven localities revealed a weak but significant genetic structure partially explained by geographic distance. None of the landscape models had a significant effect on genetic structure over that of the Euclidean distance alone and no evidence for efficient barriers to dispersal was found. Contemporary gene flow was not severely limited for *A. sapidus* as shown by high migration rates estimates (>10%) between non-neighbouring areas. Sex-biased dispersal tests did not support differences in dispersal rates, as shown by similar average axial parent-offspring distances, in close agreement with capture-mark-recapture estimates. As predicted, our results do not support any preferences of the species for specific landscape attributes on their dispersal pathways. Here, we combine field and molecular data to illustrate how a habitat specialist mammal might disperse like a habitat generalist, acquiring specific long-distance dispersal strategies as an adaptation to patchy, naturally fragmented, heterogeneous and unstable habitats.

## Introduction

Animal dispersal is commonly defined as the movement of individuals away from their home ranges with no subsequent return (at least, temporally) [1]. Although the decision of *how*, *when* and *where* to disperse is taken by individuals, its consequences extend to population and species levels. Individuals disperse as an effective strategy for the avoidance of inbreeding, resource competition, and kin competition [2], and this initiates important ecological and genetic feedbacks in spatially structured populations [3]. It has been classically debated whether patchy distributions of species result from pure distance effects (i.e. individuals mostly recruiting near their parents) [4], species-specific environmental responses [5] or the interaction of these two, which might depend on the scale at which the study is conducted [5], [6]. In naturally or anthropogenically fragmented landscapes, the degree of fragmentation and the spatial configuration of the network of patches will influence dispersal routes and probabilities and, consequently, will affect the rates of colonization of empty patches and the distribution of genetic diversity [7]. These consequences make of dispersal a keystone process in ecological and evolutionary studies. In this sense, dispersal may be seen as the glue that holds populations connected, but also as the *glue* that connects different scales and disciplines [8].

Gene flow is one of the important consequences of *effective* dispersal (i.e. when it is followed by breeding success) and is expected to homogenize the genetic variation among populations and counteract the structuring effects of drift. Therefore, species might show strong genetic structure when gene flow among populations is reduced, either because the geographic distance exceeds average dispersal distance or because effective barriers (or filters) to dispersal separate the populations. Genetic structure will thus be greater for low mobility than for highly mobile species at a particular geographical scale. Classical analyses of patterns of gene flow have usually addressed their extent and distance components, often revealing a monotonic decrease of gene flow with distance (isolation-by-distance), where geographic distance is calculated as the Euclidean distance separating individuals or populations. This approach implicitly assumes that dispersing individuals travel in a straight line across a homogeneous or irrelevant landscape matrix. A more recent approach has highlighted the relative importance of the landscape matrix heterogeneity on the dispersal behavior of species, by showing a better correlation of gene flow with landscape-modified distances than with purely Euclidean distances [9].

Species are often classified into habitat generalist or specialists based on habitat requirements: while the former can exploit multiple habitat types or food sources, the latter are restricted to only one or few habitats. Like habitat generalists, specialists in large and continuous habitats can move rather freely across space, rendering populations with reduced spatial and genetic structure. Quite often, however, habitat specialists are restricted to more or less scarce and scattered patches of suitable habitat embedded in an unsuitable habitat matrix. Given that small and isolated populations have increased risks of extinction, highly specialized species inhabiting patchy habitats require a high ability to move through the matrix in order to avoid genetic and demographic isolation [10]: paradoxically, habitat specialists must behave as dispersal generalists. Generalist dispersal patterns have been described in plants [11], [12] and invertebrates [13] occupying scarce and patchy habitats, although this possibility has not been yet assessed in mammals.

We set out to test this prediction using southern water vole (*Arvicola sapidus*) as a case study of a species tightly associated to naturally fragmented habitats embedded in heterogeneous but largely hostile habitat matrices (see below). We first estimate gene flow among the populations of this rodent in the study area through indirect and direct approaches based on neutral autosomal microsatellite genotypes and mitochondrial control region sequences. We will then evaluate the relative role of the landscape matrix in shaping gene flow patterns through several landscape genetic approaches. According to our prediction, a high ability of southern water voles to disperse must be reflected on a weak genetic structure and minor effects of landscape on gene flow patterns (i.e. isolation-by-distance pattern more pronounced than any other landscape-based model). Finally, we will try to elucidate whether the observed genetic structure is a result of sex-biased dispersal, as found for many mammals. Patterns of dispersal inferred from genetic markers will be compared to those obtained in previous field studies [14]. Our results suggest that Southern water vole may have acquired generalist habitat choice for dispersal and/or the ability for long-dispersal as a response to patchy, naturally fragmented and heterogeneous habitats.

## Results

### Microsatellite diversity and structure

A total of 142 individuals distributed in seven sampling areas throughout the region were genotyped using 10 polymorphic loci. All loci were highly variable, the number of alleles per locus ranging from 8 to 29. Allelic richness averaged over loci, and adjusted for the minimum sample size of 8 individuals, was between 2.889 and 10.713. Average expected population heterozygosities varied from 0.6860 to 0.7808 (Table 1). The only locus that showed deviation from HWE in all but two populations (RES2 and ROC) was AV14-2 (all p<0.05), whose moderate to high inbreeding coefficient (*F_IS_* = 0.297) reveals a deficit of heterozygotes potentially caused by null alleles. Overall *F_IS_*, which was moderate and significant when AV14-2 was included (*F_IS_* = 0.048, 95% CI [0.003, 0.114]), became low and non significant when this locus was excluded from the dataset [*F_IS_* = 0.015, 95% CI (−0.002, 0.031)]. Consequently, this locus was excluded in global tests of HWE for each population, but all loci were considered for the rest of analyses. Significant deviations from Hardy-Weinberg Equilibrium across loci (HWE, Table 1) were detected in the three populations within the Biological Reserve of Doñana (all p<0.05) (range H_E_ = 0.6860–0.7632; range H_O_ = 0.6746–0.7267), while significant heterozygote excess was found at RES1 (H_E_ = 0.6984, H_O_ = 0.7222) and ROC (H_E_ = 0.7808, H_O_ = 0.7875).

**Table 1:** Genetic diversity and Hardy-Weinberg equilibrium tests in the seven populations throughout Doñana Natural Region.

	N	AR	HWE	H(E)n.b.	H(O)
**Abalario1 (ABA1)**	23	5.269	0.0513	0.763	0.7096
**Abalario2 (ABA2)**	35	5.916	0.0819	0.7774	0.7159
**Reserve1 (RES1)**	18	4.304	0.004	0.6984	0.7222
**Reserve2 (RES2)**	15	5.477	0.001	0.7632	0.7267
**Reserve3 (RES3)**	19	4.57	0.0352	0.686	0.6746
**Marismillas (MAR)**	24	4.681	0.0518	0.7035	0.6542
**Rocina (ROC)**	8	5.582	0.8817	0.7808	0.7875

N, number of individuals; HWE, significance from Hardy-Weinberg equilibrium; AR, mean allelic richness overall loci; H(E)n.b. non–biased expected heterozygosity; H(O), observed heterozygosity.

**Figure 1:**
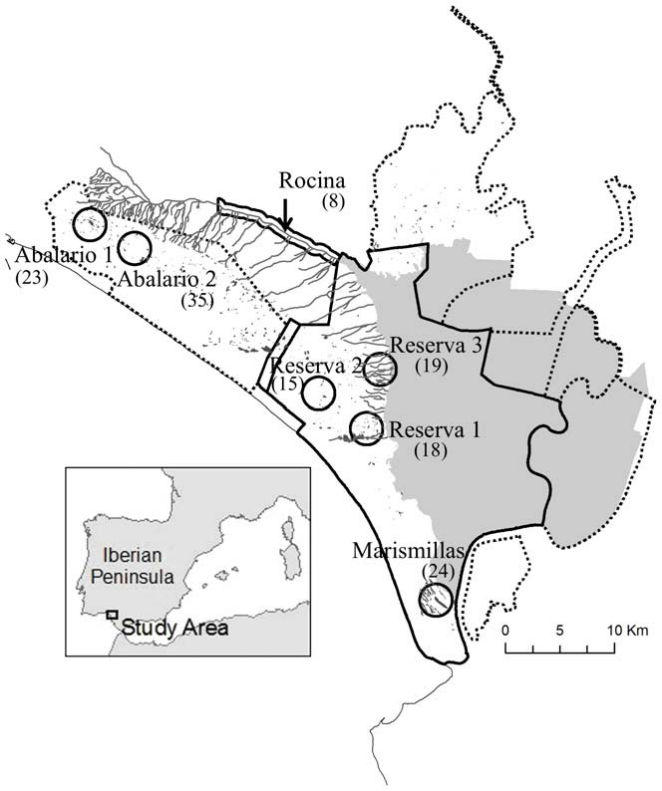
Factorial Correspondence Analysis (FCA) performed with 142 individuals trapped in Doñana.

Individuals trapped within the same geographic population tended to cluster together in FCA plots, but with some overlap between neighbouring areas (Figure 1). The distribution of the sampling areas in the FCA coincided with their geographical location (e.g. ABA1 and MAR–the most geographically separated populations-are the most separated in the FCA). This pattern is reflected in a moderate levels of genetic structure (overall *F_ST_* = 0.072, 95% confidence interval [0.058–0.089]. Exact Pairwise *F_ST_* values ranged from 0.028 to 0.116 (Table S1), being all significantly different from zero. Finally, the hierarchical and spatial analysis of genetic structure (SAMOVA) does not reveal any higher-level statistically significant population groupings, since Φ_CT_ values decrease as *k* increases beyond one.

### Mitochondrial variation

**Figure 2:**
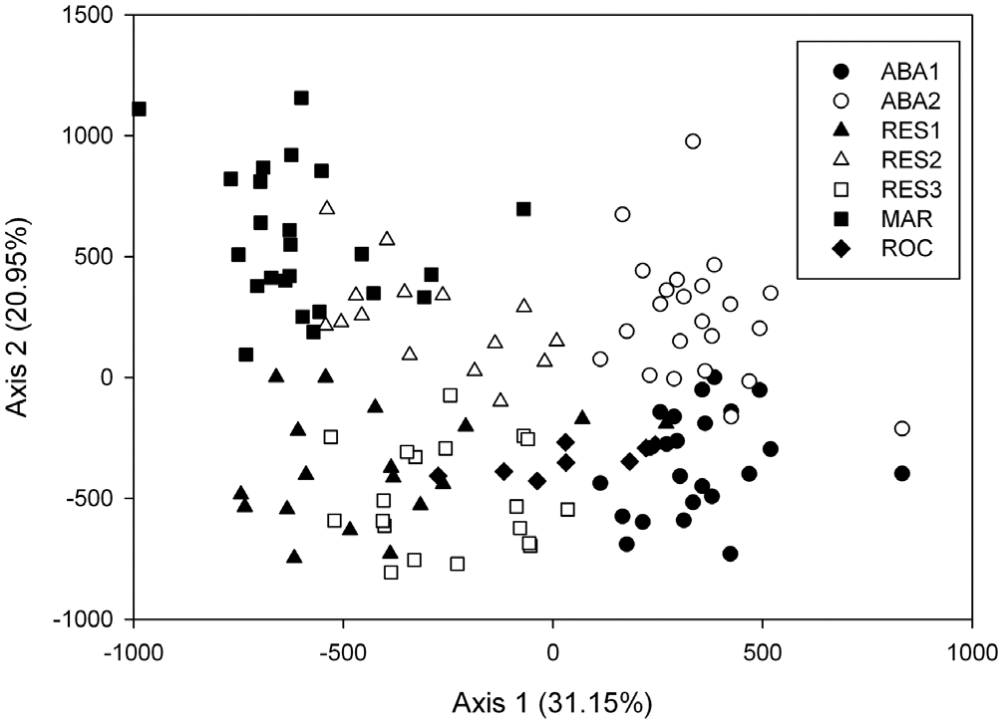
Regression of interpopulation genetic distances (*F_ST_*). Pairwise comparisons inferred with microsatellites and mitochondrial Control Region after calibration (see text) are noted with filled circles (solid line) and open circles (dashed line), respectively, on the natural logarithm of geographic distance among populations. Equation of microsatellites regression: y = 0.108+0.018x. Equation of Control Region regression: y = −0.415+0.057x.

Ten mitochondrial haplotypes defined by 17 segregating sites were found after the sequencing of a 1024 bp Control Region in 47 individuals (Table S2). Most variation was due to transitions (96.24%), with only 3.76% transversions and no indels (average number of nucleotide differences, *k* = 6.278). Nucleotide diversity (π) varied throughout Doñana from π = 0 (MAR) to π = 0.006 (RES3) (Table S2). Haplotype diversities (*H*) were slightly higher in Northern localities (maximum *H* = 0.929, ABA2). The distribution of haplotypes throughout Doñana did not show any obvious geographical pattern, although haplotypes 7 (n = 1) and 10 (n = 1) were only found in RES1 and ABA2, respectively, and four haplotypes were distributed only in the Northern region (haplotypes 1, 3, 8 and 9, located in ABA1, ABA2 and ROC) (Table S2). We found a strong mitochondrial genetic structure (overall *F_ST_*_mt_ = 0.3682, which would translate to *F_ST_*_mt_' = 0.1272 for direct comparison with microsatellites-inferred *F_ST_*). Pairwise *F_ST_* ranged from −0.0177 to 0.4717 with no significant pattern of isolation-by-distance (Figure 2; see below).

### Isolation-by-distance and landscape genetics

**Figure 3:**
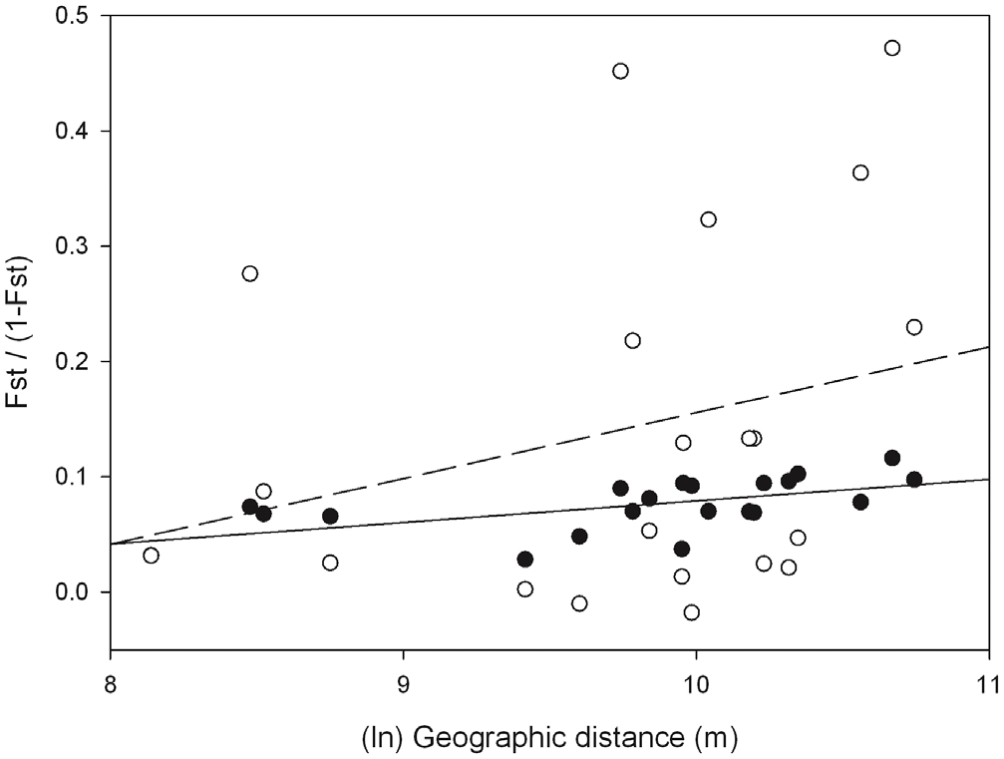
Regression of interindividual genetic distances a_r_ [**21**] between males (a) and females (b). Regression inferred on the natural logarithm of geographic distance among individuals. Equation of males regression: y = −0.084+0.031x. Equation of females regression: y = −0.098+0.027x.

Geographic (Euclidean) distance between sampled areas explained 34% of the microsatellite genetic variance (IBD, Mantel Test: r = 0.5844, p<0.01) (Figure 2). At the individual level, a significant pattern of IBD was obtained when considering all individuals (r = 0.288), as well as only males (r = 0.2830) or females (r = 0.2541) (all p<0.0001) (Figure 3).

Least Cost Distances calculated from different landscape attributes were robust to variations in the arbitrarily assigned maximum cost values (CV_POND_ = 0.096±0.004; CV_DRE_ = 0.171±0.007). LCDs based on vegetation, drainages and ponds were all positively correlated with genetic distances in Doñana and explained a slightly higher proportion of the observed genetic variance than Euclidean distances (LCD_VEG_, r = 0.5386; LCD_PONDS_: r = 0.6078; LCD_DRA_: r = 0.6089, all p<0.05). However, none of these landscape effects remained significant once the effects of Euclidean distance were discounted (all Partial Mantel test, p>0.05). Moreover, delta differences of AICc between the model with lowest AICc (Euclidean distance) and landscape modified line models were always less than two.

### Sex-biased dispersal

Observed differentiation indices were generally higher for males than for females (females: *F_ST_*  = 0.0652, mAIC  = −0.01744, vAIC  = 24.19336; males: *F_ST_*  = 0.0805, mAIC  = 0.01926, vAIC  = 14.09704), although none of sex-biased dispersal tests rejected the null hypothesis of equal dispersal between sexes (all p>0.05). The same result was obtained when only adults trapped in ABA1 and ABA2 after dispersal but before breeding were considered (*F_ST_*, mAIC and vAIC tests, all p>0.05). Finally, very similar average axial parent-offspring distances were estimated for males and females (*σ_o_* = 732, *σ_m_* = 668 m, *σ_f_* = 661 m) from the slopes of the individual-based IBD analyses (*b_o_* = 0.0203, *b_m_* = 0.0243, *b_f_* = 0.0248) and considering an effective density of 5–10 ind./km^2^.

### Contemporary gene flow

Most sampling locations in Doñana showed low recent immigration rates (Table 2), with the exception of four cases (migration rates from ABA1 to ABA2: *m* = 0.1131; from ABA1 to ROC: *m* = 0.1616; from MAR to RES2: m = 0.2199; from RES3 to RES2: *m* = 0.0456). This pattern suggests a moderate metapopulation dynamics within the study area with either low or relatively high immigration rates proceeding from nearby populations.

**Table 2:** Means and 95% confidence intervals of the posterior distributions of *m*, the migration rate into each population for the three replicates run on Bayesass.

To							
Rates from	ABA1	ABA2	RES1	RES2	RES3	MAR	ROC
**ABA1**	**0.9793**	**0.1131**	0.0086	0.0109	0.006	0.0033	**0.1616**
	(0.9253–0.9997)	(0.0013–0.2972)	(0–0.0522)	(0–0.0585)	(0–0.0332)	(0–0.0229)	(0.0259–0.2959)
**ABA2**	0.0044	**0.8526**	0.0073	0.0095	0.0039	0.0041	0.0169
	(0–0.0326)	(0.6749–0.9830)	(0–0.0419)	(0–0.0467)	(0–0.0257)	(0–0.0228)	(0–0.0792)
**RES1**	0.0031	0.0065	**0.9487**	0.0186	0.011	0.0036	0.0317
	(0–0.0206)	(0–0.0379)	(0.8768-0.9937)	(0–0.0869)	(0–0.0319)	(0–0.0232)	(0.0001–0.1184)
**RES2**	0.0036	0.0064	0.0051	**0.686**	0.004	0.005	0.0179
	(0–0.0262)	(0–0.0361)	(0–0.0323)	(0.6671–0.7326)	(0–0.0246)	(0–0.0300)	(0–0.0807)
**RES3**	0.0032	0.0078	0.0083	**0.0456**	**0.9656**	0.0033	0.0545
	(0–0.0231)	(0–0.0443)	(0–0.0482)	(0.0031–0.1237)	(0.9076–0.9993)	(0–0.0202)	(0–0.2109)
**MAR**	0.0031	0.0065	0.0175	**0.2199**	0.0048	**0.9773**	0.0167
	(0–0.0226)	(0–0.0389)	(0–0.0692)	(0.1186–0.2997)	(0–0.0333)	(0.9318–0.9985)	(0–0.0830)
**ROC**	0.0034	0.0071	0.0045	0.0095	0.0047	0.0034	**0.7008**
	(0–0.0259)	(0–0.0422)	(0–0.0264)	(0–0.0475)	(0–0.0327)	(0–0.0210)	(0.6678–0.7791)

The populations from which each individual was sampled are listed in the columns, while the populations from which they migrated are listed in the rows. Values along the diagonal are the proportions of individuals derived from the source populations in each generation. Most recent migration rates estimates are low, except for those in **bold**. 95% confidence intervals are large but substantially smaller than those obtained in simulations where there is no information in the data [non-migration rates: 0.833 (0.675–0.992); migration rate: 0.0277 (1.15E-07, 0.144), indicating our dataset contains enough information to suitably estimate recent migration.

## Discussion

### Dispersal of Arvicola sapidus

Our results provide the first insight into the genetic structure and patterns of gene flow of Southern water voles in a Mediterranean patchy habitat. This, together with the existing knowledge of its natural history and habitat preferences, contributes to the understanding of the patterns and significance of dispersal in the species and illustrates the potential contribution of molecular markers to the study of dispersal in small mammals.

We found a moderate level of population genetic diversity (average H_E_ = 0.74), slightly lower than that previously reported in metapopulations of European water voles (*Arvicola terrestris*) [15]. This difference is, however, slight and might be partly due to the effect of ascertainment bias. Two populations (RES1 and RES2) were not in Hardy-Weinberg equilibrium, even when the AV14-2 marker (potentially affected by null alleles) was excluded; this was due either to a significant excess (RES1) or a deficit of heterozygotes (RES2). Hardy-Weinberg disequilibrium reflects deviation from panmixia and random mating and it has been found to be common in a well studied metapopulation of aquatic *A. terrestris* in Scotland [16], but less so in a fossorial population of the same species in France [15]. Heterozygote deficit might be the consequence of a Wahlund effect due to the sampling of a few family groups [17]. Heterozygote excess reported in RES1 and ROC suggest some strategies for inbreeding avoidance, as previously described for species of the close genus *Microtus* [18], or may be the consequence of random differences in allelic frequencies between males and females facilitated by sex-biased dispersal, a pattern that we, however, did not confirm in our system (see below).

Overall mitochondrial nucleotide and haplotype diversities in the area were high, but highly variable among local SWV populations, including an absence of diversity in the southernmost population (MAR). Low levels of haplotype diversity might be reflecting recent founder or bottleneck effects affecting females and absence of recent gene flow by females (but see below). Besides, we found a moderate mitochondrial genetic structure reflecting significant differences in haplotype frequencies, but without an obvious geographic pattern (Table S2). Furthermore, no significant pattern of isolation-by-distance was found for mitochondrial variation, although this might be attributed to low power deriving from small sample sizes. Overall, mitochondrial patterns are indicative of a non-equilibrium situation, which is only partially supported by nuclear data (see below).

In our study, we have investigated dispersal and nuclear gene flow using two complementary approaches that provide estimates at two temporal scales and whose comparison allows us to address the existence of migration-drift equilibrium and the temporal dynamics of gene flow. Direct estimates of recent gene flow are clearly bimodal, with most pairwise estimates being low (mean 0.06%) and significant migration rates from ABA1 to ABA2, ABA1 to ROC, MAR to RES2 and RES3 to RES2, suggesting directional and simultaneous or recurrent events of dispersal between the same two populations. All four cases of recent migration occur between nearby (but not always neighboring) populations and are clearly asymmetrical, identifying a source and a recipient population. For example, ABA1 appeared as a recent source of immigrants into ABA2 and ROC, RES3 into RES2 and MAR into RES2 (Table 2). While the first three observations are between neighbouring areas, the fourth one involves two non-neighbouring areas that are in fact separated by the presence of sand dunes, *a priori* a putative barrier for dispersal of SWV (the latter is also supported by shared mitochondrial haplotypes between RES2 and MAR). This pattern may result from a single or few correlated events of migration and illustrates the inherent stochasticity associated to demographic events under a metapopulational dynamic at the spatial and temporal scales considered. Furthermore, the observed patterns might be justified by recent experimental studies of dispersal in *Arvicola terrestris* in Scotland, where it was shown that juveniles disperse to distant patches making temporary stops at suitable sites between dispersal movements (“stepping-stone”) [19].

In contrast, population structure (and, presumably, average levels of long-term gene flow) seems to follow an isolation-by-distance pattern across the region, as expected under migration-drift equilibrium in a stepping-stone model. Both inter-individual and inter-population genetic distances increase linearly with geographic distance (see fig.3 and Table S1) and the placement of individual genotypes and populations in FCA plots neatly captures their relative geographic locations. No higher level grouping of populations seems to explain a significant amount of the genetic variance in SAMOVA analysis, indicating little effect of geographical barriers or demographic factors in separating groups of populations. In contrast, Berthier *et al.* (2005) [15] found genetic disruptions that were associated with both sharp relief and transition between an area of low abundance and another of high abundance in fossorial water voles in France. The Doñana area is mostly flat and water voles distribution is more or less homogeneous at a regional scale so these effects were not anticipated.

Quantitatively, the level of genetic structure observed for microsatellite markers is moderate and intermediate between those found for the fossorial *A. terrestris* in an area of grassland and open forests in France (global *F_ST_*: 0.032) [15] and in a coastal drainage system in Scotland (global *F_ST_*: 0.09) [20], over similar and smaller spatial scales, respectively. It must be noted, though, that *F_ST_* values reported above were estimated using different but overlapping sets of loci, sharing five and eight loci with those used in this study ([15] and [20], respectively), and that comparisons between studies using markers with different levels of heterozygosity must be taken cautiously [21]. Higher levels of genetic structure could be expected in our drier Mediterranean setting, where colonies of SWV might become relatively isolated due to reduced dispersal rates and large effective distances imposed by the inhospitable landscape matrix. According to our expectations, however, gene flow does not seem to be severely limited for *A. sapidus* in the Doñana region (overall *F_ST_* = 0.072 and significant IBD pattern). Genetic estimates of average dispersal distance (*σ_males_* = 668 m, *σ_females_* = 661 m) do not differ from the average distances estimated for each sex using capture-mark-recapture analyses (males, 838 m; females, 695 m; [14]). It must be noted, though, that both ecological and genetic estimates of dispersal distances might be biased [22], [23], but concordance between the two estimates is reassuring. These distances grossly match those observed in radio-tracked juvenile European water voles dispersers (average 553 m, range 159–1800 m; [19]). We could not reject the null hypothesis of similar dispersal rates for both sexes based on microsatellite markers (sex-biased dispersal tests, all p>0.05) although these methods might be biased when dispersal rates strongly differ between sexes [24]. Moreover, sex-biased dispersal analyses might be also biased by sampling dispersers born in close but unsampled populations (hence, genetically similar to the population of capture but not included in our study) [25]. However, overall genetic differentiation for the female-transmitted mitochondria was about twice that observed for biparentally transmitted nuclear microsatellite markers, even after correction for differences in Ne between nuclear and mitochondrial genomes, what would agree with the female-bias in dispersal rates observed in field studies in Doñana (dispersal rates per sex: males, 14.4%; females, 3.5%) [14]. Measures of dispersal obtained in the field must be taken with caution and only as lower interval values of dispersal, due to possible biases induced by high mortality rates and extremely complex population dynamics. Ecological and genetic estimates of dispersal rates might also differ because only the latter depends on the breeding success of individuals. Consequently, in polygynous species (as *A. sapidus*) lower *dispersal rates* in females may be compensated by their higher rates of survival and reproduction, resulting in similar effective *dispersal distances* for both sexes.

Long-distance dispersal in both sexes [16], as well as inter-sexual social attraction [19], might also explain why water voles distributed in Doñana persist with high genetic variability, even when the global population dynamic is clearly metapopulational. The observation of higher rates of female *A. terrestris* dispersal in more fragmented and unstable settings seems to support the prediction that female dispersal may be an adaptation to patchy unstable habitats, as females can improve their success by prospecting for suitable habitat when turnover rates in local patches are high [16].

### Dispersal in habitat specialists

Habitat specialists inhabiting scarce and scattered habitat patches must develop dispersal abilities to move through hostile matrices and colonize distant and/or empty patches, in order to avoid very small population sizes, reduced genetic variability and high extinction risks. Although occasionally small mammals may disperse by passive strategies [26], it is more likely for a habitat specialist living in patchy environments to have relaxed habitat requirements during dispersal [10]. For example, Telfer *et al.* (2001) [27] suggested that *A. terrestris* might show overland instead of waterway dispersal and Telfer *et al.* (2003) [28] reported long-distance dispersal of both sexes independently of landscape for a similar metapopulation. This hypothesis on dispersal in habitat specialists has received recent support from field studies (radiotracking) [19]. But, what does *overland* mean? A plentiful literature distinguishes habitat (used patches) from non-habitat (matrix) when a classification of land uses for species is intended. This binary division assumes the permeability of patches and the reluctance of individuals to cross inhospitable habitat matrices (barriers). There are, however, many species that apparently differentiate at least three kinds of habitat types: breeding habitat, dispersal habitat and the intervening landscape matrix [29] , being only the latter considered as a barrier for individual movements.

After a few failed attempts to monitor radio-collared individuals, our genetic approach has improved our knowledge of dispersal habits in SWV. Ponds, drainages and vegetation cover apparently explained the genetic structure in SWV better than Euclidean distances (see Mantel tests) but none of the landscape models were significantly better than a model based on Euclidean distance alone. Accordingly, our results do not support any preferences of SWV for specific landscape attributes on their dispersal pathways for the establishment of breeding territories.

It must be noted, though, than even when our sampling was widespread throughout Doñana, both the spatial distribution of landscape variables and the limited resolution of the landscape tessellation resulted in least cost distances that were highly correlated with Euclidean distances, what may have limited our power to detect any landscape effects. However, although landscape tessellation may not be appropriate for the study of dispersal in SWV at local scales (e.g. within geographic –sampling- populations in this study), high gene flow between non-neighbouring areas (hence, exceeding cell size) may justify its suitability for the study of dispersal through the overall region of Doñana. Therefore, considering this limitation and the fact that models incorporating landscape attributes performed slightly better in explaining genetic variance between populations, the influence of landscape in SWVs movement and dispersal cannot be completely discarded. No preference for grassland over non-grassland could be demonstrated for *A. terrestris*, using a similar approach, although in this case the landscape model explained less of the genetic variance than the null Euclidean model [15].

One of the main questions addressed by ecological studies of dispersal is *where* and *when* individuals choose to stop dispersing and settle their new breeding territory [see [3] for a review]. Behavioural ecology and landscape genetics are also trying to unravel *how* individuals move from the patch of origin to the targeted area (i.e. dispersal pathways). Can these three questions (*where*, *when* and *how*) be answered by a single factor: the natal/breeding habitat preference of individuals? [30]. Interestingly, Sacks and colleagues showed that in a habitat generalist species (coyotes, *Canis latrans*) the tendency of individuals to disperse into habitats similar to their natal habitats (i.e. natal habitat biased dispersal) results in strong genetic structure among nearby populations (i.e. although the species is considered as a habitat generalist, individuals and populations may behave as habitat specialists). In our work, we have found just the opposite pattern: a habitat specialist (southern water vole) showing genetic and dispersal patterns more related to generalists (relatively low levels of genetic structure, isolation-by-distance patterns and scarce influence of landscape on dispersal pathways).

### Conclusions

To summarize, we would like to emphasize different aspects of dispersal of SWV and their relevance for the study of general patterns of dispersal in other species. First, the respective biases on field and genetic-based estimates of dispersal might be solved by the combination of both approaches. At a local and short-term scale, *A. sapidus* dynamics in Doñana region is typically metapopulational, being dominated by frequent extinction-recolonization events of particular habitat patches (ponds). This dynamic seems to be reflected in highly variable estimates of recent gene flow and lack of genetic equilibrium at local scales. However, high dispersal rates over large distances relative to interpatch distances, seem to effectively buffer population dynamics, resulting in genetic patterns closer to genetic equilibrium at a regional and longer-time scales, more similar to those found in continuous populations. We suggest that this habitat specialist species might behave as generalist habitat species in terms of habitat choice for dispersal and/or specific long-distance dispersal strategies as a response to a patchy, naturally fragmented heterogeneous and unstable habitats.

## Materials and Methods

### Ethics statement

The authors manipulated and marked southern water voles approved by the Junta de Andalucía Consejería de Medio Ambiente and the Estación Biológica de Doñana under permits linked to project BOS2001-2391-C02-01.

### The species and the study area

The southern water vole (SWV) (*Arvicola sapidus*) (Cricetidae, Rodentia) is a habitat specialist, being exclusively associated to small vegetation patches on muddy soil along the border of water bodies, and using high vegetation cover as refuges against predators during drought periods [14], [31]. It diverged from *A. terrestris* (recently named *A. amphibius*) *ca.* 250,000 years ago [32] and has been affected by episodes of isolation in glacial subrefugia throughout Iberia and central and southern France [33]. Whereas *A. terrestris* might acquire fossorial habits in mountain regions of Europe (nearly half the size of the aquatic forms of the species) there are only aquatic habits on SWV.

Aquatic forms of both species share similar ecologies, with high reliance on waterways, small colony size and a metapopulation structure [16], whereas fossorial populations have cycle dynamics and may only show a metapopulation structure at the lowest phase of the cyclic population fluctuations [34].

**Figure 4:**
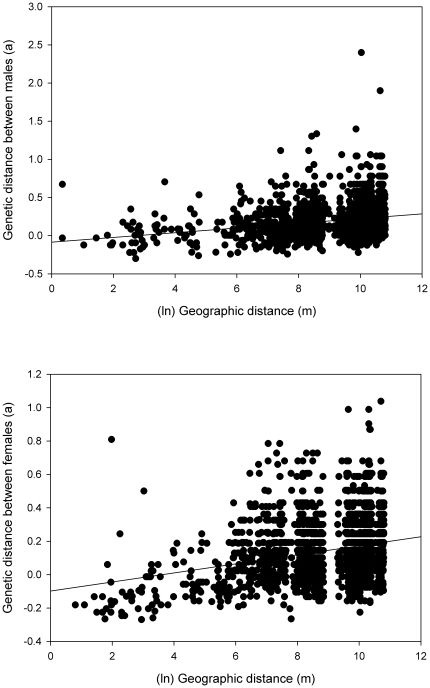
Study area at the Natural Region of Doñana. The distribution of water bodies (ponds and drainages) and the seven sampling areas where Southern water voles were trapped (circles and the stream of Rocina) are shown. Numbers in parentheses refer to sample size per sampling area. The limits of the Doñana National and Natural park are shown as a solid and dashed black lines, respectively. Marshland (flood zone) is delimited by a shaded area.

As landscape genetics studies require a rather fine-grained knowledge of the study area, we will describe with some detail the study area at the Doñana natural region (Southwest of Spain, 37° 10′ N, 6° 23′ W) (Figure 4). The climate is Mediterranean subhumid, with hot and dry summers and mild and wet winters. The average annual precipitation is about 600 mm, although there is a high interannual heterogeneity, characterized by cycles of several dry years (i.e. rainfall about or under 300 mm) interspersed with wet years (i.e. annual rainfall around or above 900 mm). Rainfall completely stops during summer. The study area (600 km^2^) is flat and mostly near sea level. There are three predominant ecosystems (from coastline to inland: mobile dunes, fixed dunes and marshes) that determine the distribution and composition of vegetation all over the region. The stream of La Rocina and its tributaries overwhelm the northern portion of the area, representing a continuous potential habitat for SWV. On the other hand, there are more than 600 water bodies throughout the fixed dunes ecosystem, whose shape varies and whose size ranges from 0.02 to 33 ha. Most small and some large ponds tend to dry out during summer, and create a network of temporary water bodies, where colonies of SWV are generally located. Bank vegetation varies and this will condition the amount of food resources and shelter available for SWV. The large and continuous marshes were probably occupied by SWV in the past, but competition with brown rats (*Rattus norvegicus*) has recently restricted SWV to the ecotone with fixed sand dunes [14]. Overall, less than 2% of the study area is considered as suitable habitat for the permanent presence of SWV. As we approach to the marshland, the phreatic table is closer to ground surface, which usually is partially flooded in winter and covered by hygrophitic shrub. So, we consider that resistance to movement of SWV should be lower (i.e. dispersal easier) closer to the marsh.

Colonies of SWV in Doñana are linked to the vegetation associated to the perimeter of the streams and water bodies. The number of adult SWVs per occupied colony per year oscillates between 6 and 31 (19.2±8.18, mean ± SD) [14], although there is high intraannual variance due to the reproductive cycle and the contingency of dispersal and colonization processes. During rainy seasons individuals breed and disperse, whereas during drought periods movements of SWV are reduced within their patch (water bodies or associated vegetation) and they do not disperse until the next breeding (rainy) season [14], [31]. A previous study has reported two reproductive maxima (autumn and spring) interrupted by a complete stop in reproduction during summer [14], [31].

The fragmentation and instability of suitable habitat patches for SWV have favoured a metapopulation dynamics with frequent extinctions and colonizations of individual ponds [average seasonal extinction rate per year: 0.24 (range: 0–0.33)] (J. Román, unpublished data). Field studies in the area have suggested a sex-biased dispersal (dispersal rates per sex: males, 14.4%; females, 3.5%) and rather long average dispersal distances (males, 838 m; females, 695 m), although the limitations of ecological methods and the limited scale of the study area may have impeded an accurate estimate of dispersal movements [14].

### Sampling design

The patterns of ponds occupancy by SWV in Doñana described by Fedriani *et al.* (2002) [31] were used to delimit six circular sampling areas of 3 kms in diameter (Abalario1, Abalario2, Reserve1, Reserve2, Reserve3 and Marismillas –ABA1, ABA2, RES1, RES2, RES3 and MAR, respectively-); one stream (La Rocina -ROC-) running through Doñana was also sampled (Figure 4). These seven areas are treated in this study as distinct geographic populations for the purpose of population genetic analyses. We visited and screened for SWV signs (trails, latrines and scattered feces) in 321 ponds and one stream (ROC), and finally trapped 142 individuals in 36 ponds and the stream; (mean: 2 ind/pond; range: 1–9 ind/pond), composing the total sample size for this study. Overall the study area, distances between ponds ranged between 32 and 46335 m. ABA1, ABA2, RES1, RES2 and RES3 were sampled in 2000 and 2001. Individuals from ROC and MAR were trapped in 2004. All trapped individuals were ear-punched and live-released. We also registered their UTM coordinates. Samples were stored at 4°C in 95% ethanol and EDTA 100 µM.

### Genotyping

We tested 24 pairs of microsatellite primers developed for *A. terrestris* [20], [35] and *Microtus oeconomus* [36]. Eight loci amplified directly and proved to be polymorphic in *A. sapidus*. Two more markers (AV10 and AV14) were used after primer redesign based on *A. sapidus* sequences obtained from products amplified with the original primers. These new primers are AV10-2 reverse (5′-CAAGGCTTGGAGCTTGGATA-3′), AV14 -2 forward (5′-TCCTCCCTCCCCAGCAAT-3’) and AV14-2 reverse (5′-GCAGAAGGGGGCAGATAAT-3′).

PCR products were indirectly labeled using a M13 5′ extension [37], except AV1, AV10-2 and AV14-2 for which 5′- labeled forward primers were used. Amplification reactions were performed in a 25-µL volume including 50-100 ng template DNA, 1x *Taq* buffer, 2 mM MgCl_2_ (0.9 mM for AV1), 0.25 mM dNTPs, 0.25 mM sequence-specific reverse and fluorescently-labelled M13 primers, 0.017 µM sequence-specific forward primer with M13 extension, 0.1 mg/ml BSA (Roche) and 0.5 U/ml *Taq* DNA polymerase (BIOTAQ™ DNA Polymerase, Bioline). AV1, AV10-2 and AV14-2 (those with 5′- labelled forward primers) required 0.25 mM forward and reverse primers. PCRs for all loci but AV1 involved temperature steps of 94°C for 5 min, 17 touchdown cycles 92°C for 30 s, 66°C for 30 s and 72°C for 30 s, decreasing one Celsius degree per cycle, and followed by 27 cycles of 92°C for 30 s, 50°C for 30 s and 72°C for 30 s. PCRs for AV1 were as follows: 94°C for 5 min, 34 cycles of 92°C for 30 s, 60°C for 30 s and 72°C for 30 s. All PCR reactions finished with 72°C for 5 min. PCR products were checked on a 2% agarose gel, run on an Applied Biosystems 3130×l Genetic Analyzer and scored with GeneMapper software v3.0 (Applied Biosystems).

The complete mitochondrial control region (1024 bp) was sequenced from a subset (n = 47) of the samples using primers F15708 and R92 [38], primer 5′-TCCCCACCATCAGCACCCAAAGC-3′ designed by [39] and four specifically designed internal primers whose combination yield partially overlapping fragments (F15816, 5′-ATGTTTTATCGTCCATACGTTCC-3′; F15872, 5′-AATCAGCCCATGCCTAACAT-3′; R15946, 5′-TAGCCGTCAAGGCATGAAG-3′; RCRasa 5′-AAAAACAACTCAAAATTCCAAAA-3′) [40]. PCR amplifications were performed as follows: 94°C for 5 min, 40 cycles at 92°C for 30 s, 62°C for 30 s and 72°C for 30 s, finishing with 72°C for 5 min. We also included in each set of PCR reactions positive and negative (water) DNA controls to monitor performance and contamination, respectively. 5 µL of PCR products were purified with 2 µL ExoSAP-IT enzyme (Exonuclease I and Shrimp Alkaline Phosphatase in buffer) (USB Corporation), by incubating during 15 minutes at 37°C and inactivating 15 minutes at 80°C. Sequencing reactions were performed using the Applied Biosystems BigDye® Terminator Cycle Sequencing Kit v. 1.1 following the manufacturer's instructions, and the same primers used for the amplification. Reactions were analysed in an Applied Biosystems 3130×l Genetic Analyzer. Forward and reverse sequences for each PCR product were edited and assembled using Sequencher 4.6 (Gene Codes Corporation, Ann Arbor, MI).

### Genetic data analysis

#### Microsatellite data

GENEPOP v3.4 [41] was used to test for deviation of Hardy-Weinberg equilibrium at each locus within each sampling unit. Levels of observed (H_O_) and unbiased expected heterozygosities [42] were estimated with GENETIX v4.05 [43] for each sampling unit by locus and over all loci. We found incongruent results among runs and between clustering algorithm analyses [44], [45] even once Markov chains converged and also when different statistical extensions to STRUCTURE [46] and BAPS (individual and group of individuals based analyses) were performed. This may be due to our aggregated sampling scheme and a global pattern of isolation-by-distance in the study system [47], [48]. We, therefore, discarded clustering analyses for population genetic structure inference. Instead, a Factorial Correspondence Analysis (FCA) was performed with GENETIX 4.05.2 to visualize genetic variation within and among geographic sampled areas. FSTAT v 2.9.3 [49] was used to calculate allelic richness per locus per population. Overall Wright's *F_ST_* and linearized pairwise F-statistics [*F_ST_*/(1- *F_ST_*)] were estimated with GENETIX 4.05.2 [43]. We also computed Rousset's inter-individual genetic distances (*a_r_*) [23] between 142 individuals using GENEPOP v3.4. We also performed a Spatial Analysis of Molecular Variance (SAMOVA) [50], by which sampling points (individual ponds) were grouped into *k* groups in a way that the proportion of genetic variance among groups (Φ_CT_) is maximized. We ran SAMOVA with 1000 simulated annealing processes for *k* values ranging from 2 to 10. If our sampling scale was adequate to dispersal patterns of SWV, we would expect *k* = 7 (i.e. the number of geographic populations used in this study), where each group (*k*) must include those ponds embraced by the 3 km diameter. On the other hand, *k*<7 would suggest higher gene flow than expected between separated geographic populations.

#### Mitochondrial data

Global and per population nucleotide (π) and haplotype (H) diversities, number of segregating sites (S) and average number of pairwise differences (k) were estimated with DnaSP 4.5 [51]. We calculated population pairwise *F_ST_* [52] using control region sequences as implemented in ARLEQUIN v3.0 [53]. Since *F*-statistics derived from mitochondrial and nuclear data assume equilibrium under different effective populations sizes, they need to be calibrated before comparison. For this purpose, we used the expression *F_ST_*_n_ = *F_ST_*_mt_/(4–3 *F_ST_*_mt_) derived from [54], where *F_ST_*_n_ and *F_ST_*_mt_ are *F*-statistics inferred using nuclear and mitochondrial markers, respectively. Because extensive homopolymer sequences hamper the sequencing reaction of the entire mitochondrial control region, we only sequenced a subset of 47 samples [40].

### Isolation-by-distance and landscape genetics

To calculate both Euclidean and Least Cost Distances (LCD) (see below) we used the weighted mean spatial coordinate of all individuals trapped as the geographic coordinate of each sampling area. Linearized pairwise *F_ST_* values were plotted against log-transformed Euclidean distances [55] to test for a negative correlation of genetic differentiation with geographic distance. Isolation-by-distance analyses were performed using the IBD Web Service v3.05 (IBDWS 3.05) [56]. IBDWS 3.05 estimated the normalized statistic (r) and its statistical significance after 30000 permutations using a Mantel Test.

We evaluated the effects of ponds surface, drainages length and vegetation cover on the genetic structure of SWV, habitat features previously described as determinants for the settlement of the colonies in the study area [14]. Here, Least Cost Distance (LCD) is used as the distance covered by an individual on its movement between two localities if one of the previous landscape attributes were favored. We divided vegetation cover into five different categories in a decreased order of assumed preference by water voles: aquatic vegetation, scrubland cover higher than 50%, scrubland cover between 20–50%, vegetation cover less than 20% and others (farming, buildings…). The whole region of Doñana was divided into a lattice of 500×500 m cells and the proportion of each of the landscape types estimated within each cell. We obtained a cost surface where the lowest cost values were assigned to landscape cells that maximized ponds surfaces, drainages or vegetation cover. Least cost paths were then calculated based on the cost of dispersing across each type of landscape cell with the extension PathMatrix [57] for ArcView TM3.2 (Environmental Science Research Institute, Redlands, USA). We also performed cost analyses [58] to evaluate the robustness of our results when varying the assigned cost value over a wide range of maximum values. To test for a correlation between LCD and genetic distance and between these two variables once the effect of the Euclidean distances is discounted, we used Mantel and Partial Mantel tests, respectively. Both Mantel and Partial Mantel test were performed in IBDWS 3.05. We also used the delta difference among the respective corrected Akaike's Information Criterion (AICc) as a statistical test to evaluate the goodness of fit of each model. AICc were calculated from the least-squares regressions and adding a second order correction because of the small sample size as follows AICc = 2k+n*ln(RSS/n) + [2k*(k+1)]/(n-k-1)], where *k* is the number of landscape features in the model, *n* is the number of populations and *RSS* is the residual sum of squares. Because of the non-independency of the data, no proper method to estimate AIC for pairwise data has been developed. Nevertheless, we used the number of populations as degrees of freedom instead of the number of pairwise comparisons as a better fit of Mantel's test to p-values of ordinary least squares regression has been shown [59]. When delta difference between two models is lower than two, these models do not statistically differ in explaining the variance of genetic distances between populations.

Hence, two models whose delta difference on their AICc was lower than two could be considered as equally likely [60]. The higher is the difference of delta values, the better the model with the lowest AICc. According to our null hypothesis, we would expect delta values lower than two between Euclidean line distance models and other landscape modified line models.

### Sex-biased dispersal

Sex-biased dispersal promotes differences in genetic structure between sexes. We tested differences in dispersal rates between sexes using two approaches. First, we regressed pairwise genetic distances (see above) on geographic distances using an independent datasets for males (n = 68) and females (n = 74) and compared both slopes. Second, we used FSTAT v2.9.3 to calculate *F_ST_* and an assignment index (AI) per sex and population pair; these two indices have been shown to be most powerful and least sensitive to changes in the magnitude of sex biased dispersal [24]. Lower *F_ST_* values, negative mean assignment indexes (mAIC) or larger variances of the AI (vAIC) are expected for the dispersing sex. The method proposed by Goudet *et al.* (2002) [24] is based on randomization procedures and eliminate pseudoreplication problems arising from the comparison between sexes. We also performed this analysis using a dataset composed by adults trapped in ABA1 and ABA2 before breeding (N_females_ = 14, N_males_ = 16) in order to avoid the effect caused by including predispersing individuals in the dataset.

Assuming IBD in a two-dimensional space, we can estimate the average squared axial parent-offspring distance (*σ^2^*), which can be interpreted as an average dispersal distance, using the slope (*b*) of the regression of interindividual pairwise genetic distances (*a_r_*, see above) on the geographical distances [23]. Considering 5–10 ind./km^2^ as effective density (*D*) (A. Centeno-Cuadros, unpublished data) we can estimate a sex-specific σ^2^ using their respective slope obtained in the IBD analyses [61]. Genepop v3.4 was used to infer *b* using the whole (142 individuals) and sex-filtered datasets. Lower values of *b* would result from higher gene flow and higher average dispersal distance.

### Contemporary gene flow

Evidence of recent migration events among sampled areas was assessed using the Bayesian multilocus genotyping procedure implemented in Bayesass [62]. This method does not assume migration-drift nor Hardy-Weinberg equilibrium, two common assumptions that are rarely met in species with high generational overlap and intense population dynamics. We ran three replicates of this Markov Chain Monte-Carlo (MCMC) based approach for a total of 3×10^6^ iterations to assure that chains reached the stationarity. Posterior probability distributions of migration parameters were estimated by sampling MCMC chains every 2000 iterations, after discarding the first 10^6^ iterations as burning.

## Supporting Information

Table S1 Linearized pairwise *F_ST_* distances between populations estimated with microsatellite data (all p<0.05, upper diagonal) and geographic distance (meters, lower diagonal).
(DOC)

Table S2 Haplotypes of Control Region and their geographical distribution all over the Natural Region of Doñana **(a)**. Control Region nucleotide diversity by sampling area and overall study system **(b)**. Sample size (*N*), number of segregating sites (*S*), nucleotide diversity (*π*), number of haplotypes (*h*), haplotype diversity (*H*) and mean number of nucleotide differences (*k*) are reported. Standard Deviations are given in parenthesis (*SD*).
(DOC)
