# Supplementary material for: Prisoners in Their Habitat? Generalist Dispersal by Habitat Specialists: A Case Study in Southern Water Vole (Arvicola sapidus)
Source: PLoS One. 2011 Sep 9;6(9):e24613. doi: 10.1371/journal.pone.0024613 (PMC3170359; doi:10.1371/journal.pone.0024613)
Supplement: Table S1 — Linearized pairwise FST distances between populations estimated with microsatellite data (all p<0.05, upper diagonal) and geographic distance (meters, lower diagonal). (DOC) [file pone.0024613.s001.doc]

**Table S1**

**ABA1 ABA2 RES1 RES2 RES3 MAR ROC**

**ABA1**  0.0319 0.0944 0.0700 0.0691 0.1161 0.0284

**ABA2** 3427 0.1025 0.0698 0.0961 0.0976 0.0484

**RES1** 27725 31152 0.0739 0.0679 0.0900 0.0922

**RES2** 22952 26377 4802 0.0657 0.0373 0.0701

**RES3** 26796 30176 5031 6315 0.0945 0.0812

**MAR** 42988 46335 17008 20968 21060 0.0781

**ROC** 12279 14778 21677 17727 18767 38594
