# Supplementary material for: Prisoners in Their Habitat? Generalist Dispersal by Habitat Specialists: A Case Study in Southern Water Vole (Arvicola sapidus)
Source: PLoS One. 2011 Sep 9;6(9):e24613. doi: 10.1371/journal.pone.0024613 (PMC3170359; doi:10.1371/journal.pone.0024613)
Supplement: Table S2 — Haplotypes of Control Region and their geographical distribution all over the Natural Region of Doñana (a). Control Region nucleotide diversity by sampling area and overall study system (b). Sample size (N), number of segregating sites (S), nucleotide diversity (π), number of haplotypes (h), haplotype diversity (H) and mean number of nucleotide differences (k) are reported. Standard Deviations are given in parenthesis (SD). (DOC) [file pone.0024613.s002.doc]

**Table S2**

**a)**

**Nucleotide position**

111111111124667

69003455779900772 **Sampled areas**

28462746382752683

**ABA1 ABA2 RES1 RES2 RES3 ROC MAR Hap.freq**

**Hap_1** GTACTTTCCTTCCTTCT 2 1 3 **Hap_2** .CGTC.C.TCCTT..AC 1 1 1 2 5 **Hap_3** ......C....TT.... 4 2 1 7 **Hap_4** .CG.CCCTTCC.T.... 2 2 **Hap_5** ACG.C.CTTCC.T.... 5 1 8 14 **Hap_6** ......C....TT.CT. 1 1 3 1 3 9 **Hap_7** A......A..C.T.CT. 1 1 **Hap_8** .CG.C.C.TCC.T.... 1 1 2 **Hap_9** .CG.CCC.TCC.TC... 2 1 3 **Hap_10** .CG.CCC.TCC.T.... 1 1

Sample size 8 8 5 6 6 6 8 47

**b)**

**N S π (SD) h H (SD) k**

**ABA1**  8 13 0.00391 (0.00151) 4 0.750 (0.139) 4.000

**ABA2**  8 13 0.00579 (0.00084) 6 0.929 (0.084) 5.929

**RES1** 5 14 0.00566 (0.00222) 3 0.700 (0.218) 5.800

**RES2**  6 6 0.00195 (0.00126) 2 0.333 (0.215) 2.000

**RES3**  6 14 0.00605 (0.00157) 4 0.867 (0.129) 6.200

**MAR**  8 0 0.00000 (0.00000) 1 0.000 (0.000) 0.000

**ROC**  6 11 0.00547 (0.00154) 4 0.800 (0.172) 5.600

**Overall** 47 17 0.00613(0.00026) 10 0.846 (0.030) 6.278
